# Impact of Transcutaneous Auricular Vagus Nerve Stimulation on Large-Scale Functional Brain Networks: From Local to Global

**DOI:** 10.3389/fphys.2021.700261

**Published:** 2021-08-20

**Authors:** Thorsten Rings, Randi von Wrede, Timo Bröhl, Sophia Schach, Christoph Helmstaedter, Klaus Lehnertz

**Affiliations:** ^1^Department of Epileptology, University of Bonn Medical Centre, Bonn, Germany; ^2^Helmholtz Institute for Radiation and Nuclear Physics, University of Bonn, Bonn, Germany; ^3^Interdisciplinary Center for Complex Systems, University of Bonn, Bonn, Germany

**Keywords:** evolving functional brain network, brain stimulation, network reconfiguration, network characteristics, centrality, EEG

## Abstract

Transcutaneous auricular vagus nerve stimulation (taVNS) is a novel non-invasive brain stimulation technique considered as a potential supplementary treatment option for a wide range of diseases. Although first promising findings were obtained so far, the exact mode of action of taVNS is not fully understood yet. We recently developed an examination schedule to probe for immediate taVNS-induced modifications of large-scale epileptic brain networks. With this schedule, we observed short-term taVNS to have a topology-modifying, robustness- and stability-enhancing immediate effect on large-scale functional brain networks from subjects with focal epilepsies. We here expand on this study and investigate the impact of short-term taVNS on various local and global characteristics of large-scale evolving functional brain networks from a group of 30 subjects with and without central nervous system diseases. Our findings point to differential, at first glance counterintuitive, taVNS-mediated alterations of local and global topological network characteristics that result in a reconfiguration of networks and a modification of their stability and robustness properties. We propose a model of a stimulation-related stretching and compression of evolving functional brain networks that may help to better understand the mode of action of taVNS.

## 1. Introduction

Vagus nerve stimulation (VNS) is an established method of brain stimulation in several diseases, including depression and epilepsy. Studies on invasive vagus nerve stimulation (iVNS) have demonstrated its effectiveness in these diseases (Elliott et al., 2011; Bottomley et al., 2020). However, being an invasive method it needs anesthesia and comprises surgical risks. Therefore, transcutaneous auricular vagus nerve stimulation (taVNS), a non-invasive external stimulation of the auricular branch of the vagus nerve, seems to be an interesting alternative. Efficacy of taVNS could be demonstrated for refractory epilepsy (Bauer et al., 2016; Liu et al., 2018; von Wrede et al., 2019) and depression (Hein et al., 2013; Fang et al., 2016; Kong et al., 2018; Tu et al., 2018). Good tolerability and usability have been demonstrated for taVNS in different health conditions (Redgrave et al., 2018). Non-invasiveness, reversibility and the possibility of a rapid start of therapy broaden the spectrum of symptoms and diseases that can be treated. Clinical applications and investigations span from cardiovascular and digestive system diseases (Kaniusas et al., 2019; Wang et al., 2020), insomnia (Wu et al., 2021), tinnitus (Stegeman et al., 2021), pain (Kaniusas et al., 2019), migraine (Straube et al., 2015) as well as to disorders of consciousness (Briand et al., 2020), aging (Bretherton et al., 2019), and cognitive impairment (Colzato and Beste, 2020).

Apart from more clinically-oriented questions on efficacy and safety there is a growing body on basic research that investigates the mode of action of taVNS in healthy subjects as well as in subjects with different diseases. For taVNS and iVNS similar projections to the nucleus of the solitary tract (NTS) and resembling pattern of brain activation could be shown (Ellrich, 2019). Studies on taVNS show widespread activity in expected vagal projections areas, including NTS, locus coeruleus, hypothalamus, thalamus, amygdala, hippocampus, as well as the prefrontal cortex and other widespread areas (Yap et al., 2020), though interpretation is difficult due to different study protocols and investigated subjects. Given these observations, we hypothesized that the impact of the global, apparently unspecific VNS-mediated activation of the brain can be suitably assessed with an analysis approach which makes use of EEG-derived, evolving functional brain networks (Bullmore and Sporns, 2009; Lehnertz et al., 2014). In a previous study (von Wrede et al., 2021), we could demonstrate that short-term taVNS has a topology-modifying, robustness- and stability-enhancing immediate effect on such brain networks derived from subjects with focal epilepsy. We here extend our investigations on short-term, taVNS-mediated modifications of global network characteristics beyond focal epilepsies and by considering local aspects related to possible modifications of individual network constituents.

## 2. Materials and Methods

### 2.1. Subjects

We investigated evolving functional brain networks from 30 subjects (20 females; age 18–55 years; median 31 years) with and without central nervous system (CNS) diseases. All subjects volunteered to participate and signed informed consent after being provided with written information and being given the opportunity to ask further questions. The study protocol had been approved by the ethics committee of the University of Bonn before the study has started. All experiments were performed in accordance with relevant guidelines and regulations. For subjects that received CNS medication, this was kept stable, and no activation methods (such as change in medication, hyperventilation, or sleep deprivation) were applied at least 24 h before stimulation.

### 2.2. Transcutaneous Auricular Vagus Nerve Stimulation and EEG Recording

Following von Wrede et al. (2021), we applied taVNS with individualized stimulation intensities (range: 0.5–5.0 mA, mean 2.2, SD ±1.1) for 1 h in the early afternoon while subjects underwent a continuous 3 h EEG recording. The stimulation phase (“S”; continuous stimulation of the left cymba conchae) was preceded and followed by 1-h pre- and post-stimulation phase (baseline phases “B1” and “B2”). Stimulation was carried out with two hemispheric titanium electrodes of a NEMOS device (tVNS Technologies GmbH, Erlangen, Germany) fitted in the left cymba conchae and using a common set of non-adjustable parameters (biphasic signal form, impulse duration 20 s, impulse pause 30 s, impulse frequency 25 Hz). Intensity of stimulation was adjusted individually and was raised slowly until the subject noticed a “tingling,” but no pain.

We recorded electroencephalograms (EEG) from electrode sites according to the 10-20 system and Cz served as physical reference. EEG data were sampled at 256 Hz using a 16 bit analog-to-digital converter and were band-pass filtered offline between 1 and 45 Hz (4th order Butterworth characteristic). Additionally, a notch filter (3rd order) was used to suppress contributions at the line frequency (50 Hz). We visually inspected all recordings for strong artifacts such as subject movements, amplifier saturation, or stimulation artifacts. Such data were labeled for further analyses.

### 2.3. Constructing Functional Brain Networks

We followed previous studies (Kuhnert et al., 2010; Dickten et al., 2016; Rings et al., 2019; Fruengel et al., 2020; von Wrede et al., 2021) and used a sliding-window approach to calculate a synchronization index *r*_*nm*_ [mean phase coherence (Mormann et al., 2000); see Appendix A1 for details] between phase time series from all pairs of brain regions (*n, m*) sampled by the *N* = 18 EEG electrodes. We derived these phase time series adaptively with the Hilbert transform from the respective EEG time series (Osterhage et al., 2007). Non-overlapping windows (with index *w*) had a duration of 20 s (5,120 data points), which represents a compromise between the required statistical accuracy for the calculation of *r*_*nm*_ and approximate stationarity within a window length (Osterhage et al., 2007; Kuhnert et al., 2013; Fruengel et al., 2020). The synchronization index was repeatedly shown to serve as an indicator for the strength of interactions in functional brain networks and is confined to the unit interval: *r*_*nm*_ = 1 indicates fully phase-synchronized brain regions and *r*_*nm*_ = 0 indexes no phase synchronization. For subsequent analyses, we excluded windows containing artifacts (on average 22% of windows from B1, 12% from S, and 20% from B2) and eventually associated network vertices with the sampled brain regions and network edges with the synchronization index values between any pair of vertices. This resulted in a time-dependent sequence of weighted and fully connected brain networks.

### 2.4. Scale-Dependent Characterization of Functional Brain Networks

We here utilized various graph-theoretical concepts to characterize functional brain networks on the level of single vertices and edges to the level of the whole network. Tracking the temporal evolution of these network characteristics allowed us to investigate possible taVNS-induced alterations at the local to the global network scale.

On the level of single vertices and edges, we utilized two opposing centrality concepts, for which corresponding centrality indices are available for both vertices and edges (Bröhl and Lehnertz, 2019) (see Appendix A2 for details). Path-based centrality indices and interaction-strength-based centrality indices were both shown previously to provide non-redundant information about important network constituents (Kuhnert et al., 2012; Geier and Lehnertz, 2017; Bröhl and Lehnertz, 2019; Bröhl and Lehnertz, 2020; Fruengel et al., 2020). Betweenness centrality CB is a shortest-path-based centrality index, which requires the definition of “length” of a path between pairs of vertices or between pairs of edges. Following Lehnertz et al. (2014) (and references therein), we related the length of the shortest path between pairs of vertices/edges to the sum of the inverse weights of edges along this path. For a pair being connected to a same vertex/edge, we set the length to zero. In case of adjacent edges, i.e., edges connected by a single vertex, we also set the length to zero. Betweenness centrality CB measures how frequently a given vertex/edge falls on the shortest path between two other vertices/edges. A vertex/edge with a high betweenness centrality acts as a bridge between other parts of the network. Eigenvector centrality CE is an interaction-strength-based centrality index and considers the influence of a vertex/edge on the network as a whole. A vertex/edge is central if the vertices/edges connected to it are also central. In the following, we refer with CBv and CBe to the vertex and edge betweenness centrality, and with CEv and CEe to the vertex and edge eigenvector centrality.

On the global network scale, we utilized the global clustering coefficient *C* for weighted networks (Onnela et al., 2005) and the average shortest path length *L* to characterize a network's global topological properties. Moreover, we utilized synchronizability *S* and assortativity *A* to assess the network's stability and robustness properties (Arenas et al., 2008; Newman, 2018) (see Appendix A2 for details). The global clustering coefficient is a measure of the degree to which vertices in a network tend to cluster together and characterizes the network's functional segregation; the lower *C*, the more segregated is the network. The average shortest path length is defined as the average number of steps along the shortest paths for all possible pairs of network vertices and characterizes the network's functional integration; the lower *L*, the more integrated is the network. Assortativity assesses the tendency of edges to connect vertices with similar or equal properties (Newman, 2003; Bialonski and Lehnertz, 2013). If edges preferentially connect vertices of similar (dissimilar) property, such networks are called assortative (disassortative). *A* is confined to the interval [−1, 1] by definition, where positive (negative) values indicate an assortative (disassortative) network. Disassortative networks are more vulnerable to perturbations and appear to be easier to synchronize than assortative networks. The latter show a stronger tendency to disintegrate into different groups than disassortative networks. Synchronizability *S* assesses the network's propensity (or vulnerability) to get synchronized by an admissible input activation: the higher *S*, the more easily can the synchronized state be perturbed (Pecora and Carroll, 1998; Barahona and Pecora, 2002; Atay et al., 2006).

In our downstream analyses, we neglected data from the first and last 15 min of each phase in order to remove possible transient effects. Together with the artifact removal, this resulted in data from *N*_*w*_ = 70 windows from B1, *N*_*w*_ = 79 windows from S, and *N*_*w*_ = 72 windows from B2, on average.

### 2.5. Statistics

We investigated differences between network characteristics from the three phases (B1, S, B2) on a per-subject basis using the Mann-Whitney U-test (B1 vs. S, B1 vs. B2, and S vs. B2; *p* < 0.05; Bonferroni correction).

## 3. Results

In order to facilitate the analysis of possible taVNS-induced network modifications from the local to the global scale, we first investigated whether alterations can be observed for the synchronization index, which we used to define edges of our evolving functional brain networks. Given that taVNS modulates disease-related symptoms in about 30–50% of cases (Hein et al., 2013; Bauer et al., 2016) and taking into account the inhomogeneity of subjects investigated here, we did not expect to identify significant alterations on a sample level. We therefore inspected, on a single-subject level and utilizing the average $\bar{R}\left(w\right)=\frac{1}{\nu }\underset{n\neq m}{\sum }r_{nm}\left(w\right)$ over all non-redundant (ν = *N*(*N* − 1)/2) pairwise synchronization indices for each phase (*w* ∈ {B1, S, B2} denotes the window number), the following scenarios:

**a***taVNS has no effect*; in this case, there should be no significant differences between values of $\bar{R}$ from phases B1 and S (immediate effect) as well as between values of $\bar{R}$ from phases B1 and B2 (enduring effect);
**b***taVNS has an immediate effect and a short-lasting enduring effect (fast relaxation)*; in this case we expect significant difference for values of $\bar{R}$ from phases B1 and S but no significant difference for values of $\bar{R}$ from phases B1 and B2;
**c***taVNS has an immediate effect and a long-lasting enduring effect (slow relaxation)*; in this case we expect significant difference for values of $\bar{R}$ from phases B1 and S and for values of $\bar{R}$ from phases B1 and B2.

**Figure 1:**
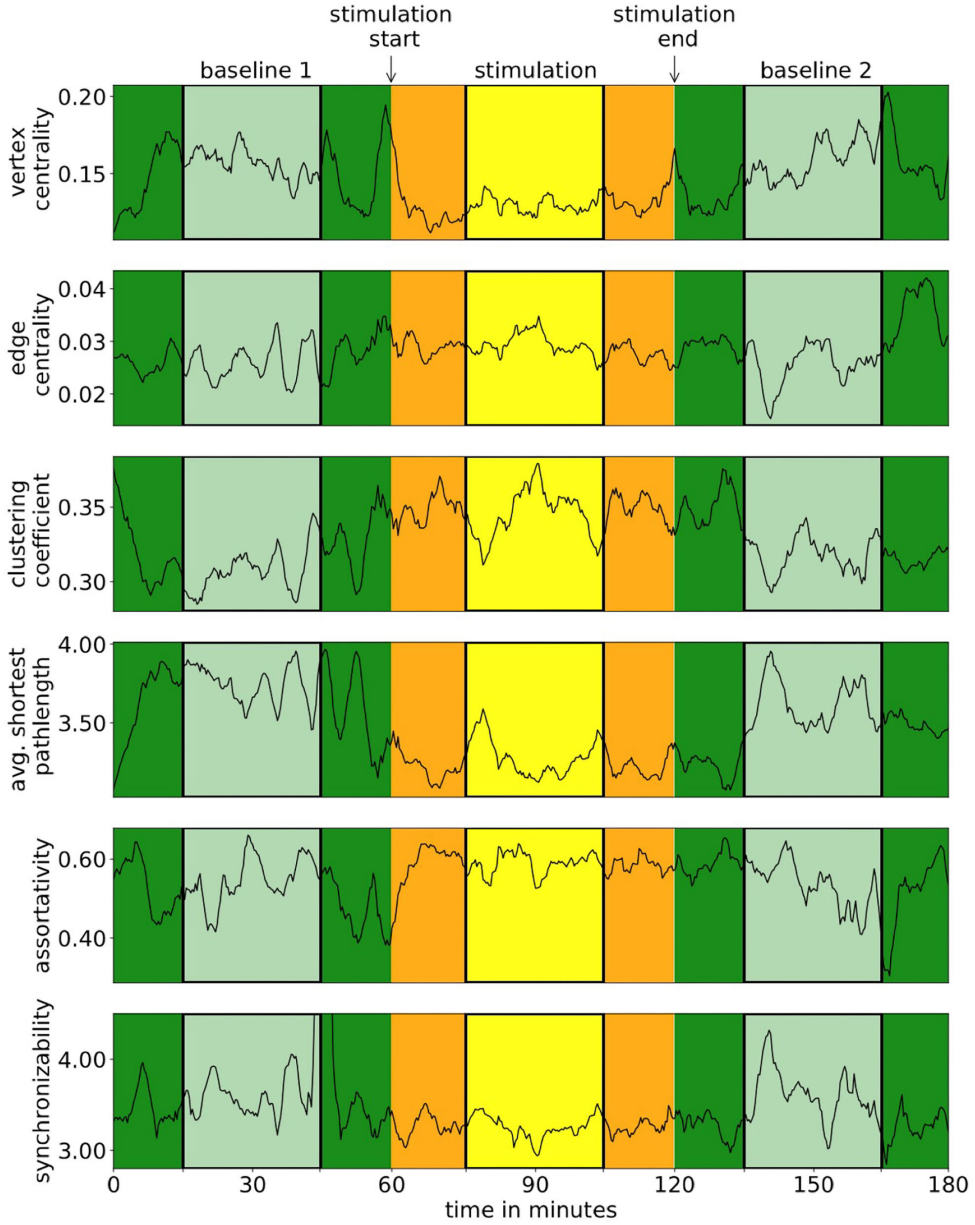
Exemplary time courses of local and global network characteristics of one subject. In our analyses, we neglected data from the first and last 15 min of each phase (darker colors) in order to remove possible transient effects.

We observed scenario **a** in seven subjects, scenario **b** in seven subjects, and scenario **c** in 16 subjects. Stimulation parameters did not differ between these groups of subjects. For all downstream analyses, we pooled the data from subjects identified in scenarios **b** and **c**. Exemplary time courses of local and global network characteristics are presented in Figure 1, and in Table 1, we report statistical moments of local and global network characteristics from phase B1 from the sample.

**Table 1:** Descriptive statistic of local (spatially averaged betweenness centralities ($\bar{C\text{Bv}}$ and $\bar{C\text{Be}}$) and eigenvector centralities ($\bar{C\text{Ev}}$ and $\bar{C\text{Ee}}$) and global network characteristics (global clustering coefficient *C*, average shortest path length *L*, assortativity *A*, and synchronizability *S*), from phase B1 for the *N*_*s*_ = 23 subjects with a taVNS-induced immediate effect and a short-/long-lasting enduring effect. For Gaussian distributed data, skewness and (excess) kurtosis would be zero with standard deviation $\sqrt{6/N_{s}}\approx 0.51$, resp. $\sqrt{24/N_{s}}\approx 1.02$.

	**$\bar{C\text{Bv}}$**	**$\bar{C\text{Ev}}$**	**$\bar{C\text{Be}}$**	**$\bar{C\text{Ee}}$**	***C***	***L***	***A***	***S***
Mean	0.03	0.22	0.01	0.07	0.34	3.30	0.41	3.05
Median	0.03	0.22	0.01	0.07	0.34	3.28	0.46	3.01
Std. dev.	0.00	0.00	0.00	0.00	0.04	0.38	0.17	0.38
Skewness	−0.47	−0.09	−0.47	0.02	0.78	−0.16	−0.51	0.60
Kurtosis	0.23	−0.94	0.23	−0.98	0.53	−0.80	−0.43	−0.15

### 3.1. Impact on Local Network Characteristics

**Figure 2:**
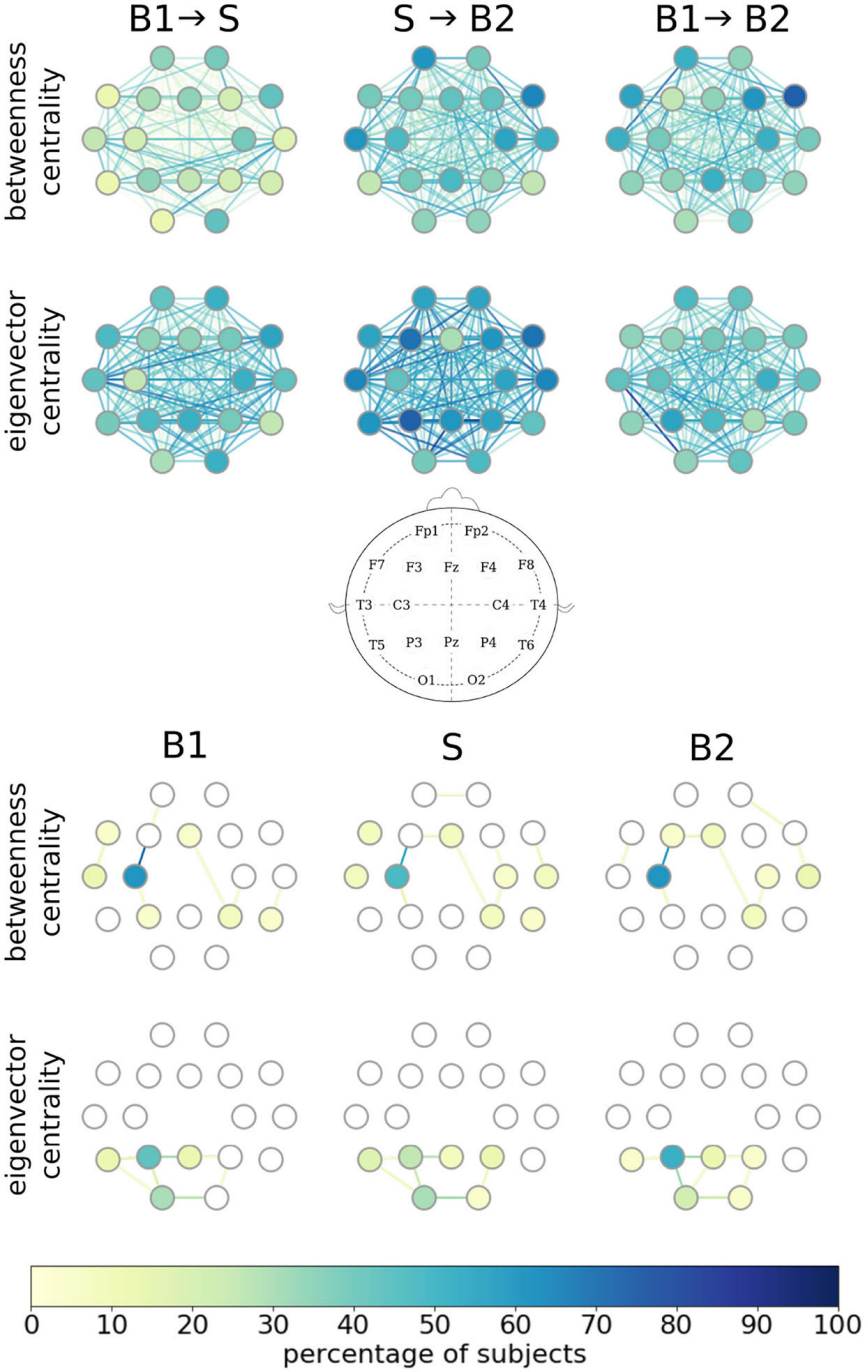
Alterations in local network characteristics when functional brain networks transit from the baseline (B1) to the taVNS stimulation phase (S) and back to the baseline (B2). Network vertices arranged according to the international 10-20 system for EEG recording (middle plot). **(Top)** Vertices and edges with significant stimulation-induced changes of their betweenness/eigenvector centralities. Color coding of individual network constituents according to the percentage of subjects for whom we obtained significant changes in vertex/edge centralities on a per-subject base. **(Bottom)** Most important vertices and edges during phases B1, S, and B2. Importance estimated with vertex/edge betweenness/eigenvector centrality. Color coding of individual network constituents according to the percentage of subjects for which the constituent was most important.

Having identified a group of subjects with taVNS-induced immediate and short-/long-lasting enduring alterations of edge weights of their evolving functional brain networks, we next investigated the impact of taVNS on other local network characteristics, namely centralities of vertices and edges. In the upper part of Figure 2, we provide a detailed picture of stimulation-related alterations of centralities of single vertices and edges. We highlight those network constituents for which we obtained significant changes in their centralities on a per-subject base. Depending on the centrality concept used, we observed some vertices and edges to exhibit taVNS-induced alterations in a higher percentage of subjects. In general, however, taVNS apparently induced alterations in centrality values of almost all vertices and edges, and no clear-cut substructures could be observed. These findings point to taVNS-induced rearrangements of the larger functional network. Since we observed, in general, a higher number of significant taVNS-induced changes with eigenvector centrality, the rearrangements within the larger functional network most likely are associated with changes in strongly connected network constituents rather than with modifications of the network's path-structure.

To examine whether taVNS impacts on the importance of individual vertices and edges, we next regarded a network constituent with the highest centrality value as most important (Lü et al., 2016) and the one with the lowest centrality value as least important (in the case of equal centrality values, we rank in the order of appearance; Liao et al., 2017). As shown in the lower part of Figure 2, different centrality concepts identified—in a high percentage of subjects—different brain regions (vertices) and functional connections (edges) between them as most important, as expected. Moreover, important edges frequently connected important vertices, and both these observations confirm previous studies (Kuhnert et al., 2012; Geier and Lehnertz, 2017; Bröhl and Lehnertz, 2019). Interestingly though, we could not identify taVNS-induced modifications of most important vertices and edges. Although this might, at first glance, contradict our findings of a large amount of constituents with significant taVNS-induced changes in their centrality values, these globally observed alterations not necessarily affect parts of the importance hierarchy of vertices and edges, such as the most important ones.

Summarizing, we observed characteristics of individual network constituents to be affected by taVNS but without a discernible spatial pattern of specific brain regions or of interactions between brain regions. This observation appears to be in line with the popular view that VNS leads to a rather unspecific, global activation of various brain structures. Interestingly, our findings also indicate that global taVNS-induced effects are constraint by the maintenance of the structure of shortest paths in a majority of subjects between the pre-stimulation phase and the stimulation phase (B1 → S). This—in combination with the changes seen for interaction-strength-based local network characteristic—hints at a possible mechanism describing the effects of taVNS.

### 3.2. Impact on Global Network Characteristics

**Figure 3:**
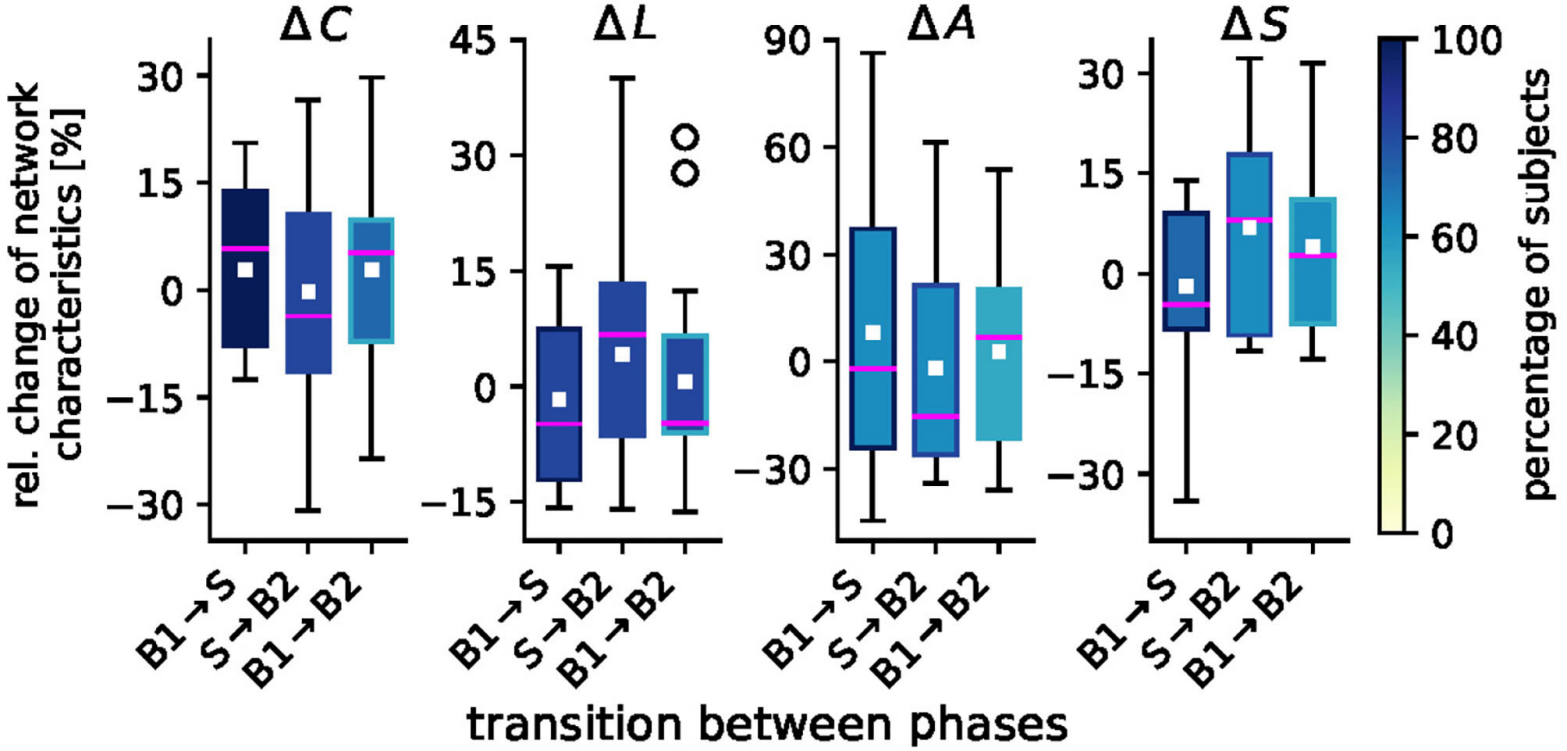
Sample distributions of taVNS-related alterations in global network characteristics (global clustering coefficient *C*, average shortest path length *L*, assortativity *A*, and synchronizability *S*). Boxplots of significant relative changes in network characteristics Δ = (*M*_*l*_ − *M*_*k*_)/*M*_*k*_, where *M*_*k*_ and *M*_*l*_ denote placeholders for the temporal average of the respective characteristics from phase *k* and phase *l*. Bottom and top of a box are the first and third quartiles, and the magenta band and the white square are the median and the mean of the distribution. The ends of the whiskers represent the interquartile range of the data. Outliers are marked by a ° sign. Color coding of boxes according to the percentage of subjects for whom we obtained significant changes in global network characteristics on a per-subject base.

We proceed with investigating the impact of taVNS on the global network characteristics global clustering coefficient, average shortest path length, assortativity, and synchronizability. In Figure 3, we provide a detailed picture of relative stimulation-related changes of these characteristics for networks transiting between the different phases. We show and report changes that were statistically significant on a per-subject base and note that—depending on the characteristic under investigation—not all of the initially selected 23 subjects presented with significant taVNS-induced alterations. We report the size $N_{s}^{\prime }$ of the respective subgroups in the following.

For the global clustering coefficient *C*, we observed medians to increase from the pre-stimulation baseline B1 to the stimulation phase S by 5.8% ($N_{s}^{\prime }=22$) and to decrease when networks transit back to the post-stimulation baseline (S → B2: −3.6% ($N_{s}^{\prime }=19$). We observed a slight overshoot effect between the pre- and post-stimulation phase [B1 → B2: 5.3% ($N_{s}^{\prime }=17$)]. We can derive similar indications with changes of the average shortest path length *L*, for which we attained a similar though inverted patterning as with *C* (which is to be expected given the definition of a path length in a weighted network); B1 → S: −4.9% ($N_{s}^{\prime }=19$); S → B2: 6.8% ($N_{s}^{\prime }=18$), and B1 → B2: −4.7 % ($N_{s}^{\prime }=19$), where the latter would indicate a slight undershoot effect. For assortativity *A*, we observed an only minor stimulation-induced decrease [B1 → S: −2.1 % ($N_{s}^{\prime }=12$)]. The tendency toward a less assortative network, however, was even increased when networks transited back to the post-stimulation phase [S → B2: −14.2 %($N_{s}^{\prime }=14$)]. As with *C*, we observed a slight overshoot effect between the pre- and post-stimulation phase [B1 → B2: 6.7% ($N_{s}^{\prime }=11$)]. We note though, that these alterations could be observed in only about half the number of the initially selected 23 subjects. For synchronizability *S*, we observed relative changes that mostly compared to the ones seen for the average shortest path length *L* [B1 → S: −4.6% ($N_{s}^{\prime }=17$); S → B2: 8.0% ($N_{s}^{\prime }=15$)]. Between the pre- and post-stimulation phase, we noted a minor overshoot effect [B1 → B2: 2.7% ($N_{s}^{\prime }=14$)].

Summarizing our findings obtained for the global network scale, we conclude that taVNS indeed induced global modifications of evolving functional brain networks in tandem with the local modifications described above. These global modifications were a reorganization of the networks' topologies, where the networks' segregation was reduced during stimulation in comparison to the pre-stimulation phase while the networks' integration was increased. As with the observed local modifications, this indicates a global activation of various brain structures, that—while spatially unspecific on the local scale—modified the topology of evolving functional brain networks and (indirectly) their stability and robustness properties in a discernible pattern.

## 4. Discussion

We investigated whether short-term transcutaneous auricular vagus nerve stimulation (taVNS) induces measurable immediate modifications of evolving functional brain networks, from the local to the global scale. In what follows, we will discuss our findings and relate them to the state of the art.

### 4.1. Modifications on the Local Network Scale

On the local scale of single vertices and edges, we observed taVNS to induce significant but unspecific modifications of local network characteristics (edge and vertex centralities) throughout the network. As a result, network constituents identified as most important during the pre-stimulation phase remained unaffected during the stimulation and the post-stimulation phase. As regards most important vertices from the pre-stimulation phase, our findings are in line with previous observations that reported left frontocentral brain regions to be most important with betweenness centrality (van den Heuvel and Sporns, 2013; Jin et al., 2014; Makarov et al., 2018) as well as parieto-occipital brain regions to be most important with eigenvector centrality (Lohmann et al., 2010) and closeness centrality (van den Heuvel and Sporns, 2013; Baravalle et al., 2019) during a so-called resting state condition. Together, these findings corroborate the common perspective of different centrality concepts generally identifying different constituents as most important (Lü et al., 2016; Bröhl and Lehnertz, 2019). Our findings are also in line with previous observations of most important edges connecting most important vertices, which has been shown to be rather typical in dense networks (Bröhl and Lehnertz, 2019).

### 4.2. Modifications on the Global Network Scale

On the global network scale, we observed taVNS to modify topological and stability- and robustness-associated network properties in a majority of subjects. In addition, we found these modifications to be enduring even after the end of the stimulation in a sizable subset of subjects (see scenarios **b** and **c**) corroborating results from a previous study (von Wrede et al., 2021). Topological network characteristics indicated on average a small stimulation-induced immediate increase in integration and a decrease in segregation of the subjects' evolving functional brain networks. After the end of the stimulation, this effect was enduring. This result contrasts findings from the study by von Wrede et al. (2021), where the same enduring effect was observed, but the immediate effect of the stimulation perceived to be reverse (in median a decrease in integration and a increase in segregation). For stability- and robustness-associated network characteristics, we here observed almost no immediate change in assortativity, i.e., in the vulnerability to synchronize. On the other hand, in the post-stimulation phase we observed an enduring effect of an increased robustness against synchronization when compared to the pre-stimulation phase. Changes in synchronizability indicated an immediate increase of the stability of a potential synchronized state of the networks during taVNS and a more easily perturbed synchronized state after the stimulation. Again, the enduring effect on stability- and robustness-associated network characteristics was comparable to findings from von Wrede et al. (2021) and the immediate effect was different. In the previous study, the immediate stimulation-induced effect also indicated a decreased vulnerability to synchronize and a decreased stability of the synchronized state. The discrepancies between the current and the study by von Wrede et al. (2021) are possibly the result of our larger group of subjects (30 vs. 14) and the inclusion of subjects with a broader range of different pathologies and etiologies, which also may assist in explaining the larger inter-subject variability of modifications of global network characteristics observed here. Further studies might shed light on the existence of different subgroups of subjects and the influence of such subgroups on taVNS-induced modifications of topological and stability- and robustness-associated network properties.

### 4.3. A Model for taVNS-Mediated Modifications of Functional Brain Networks

**Figure 4:**
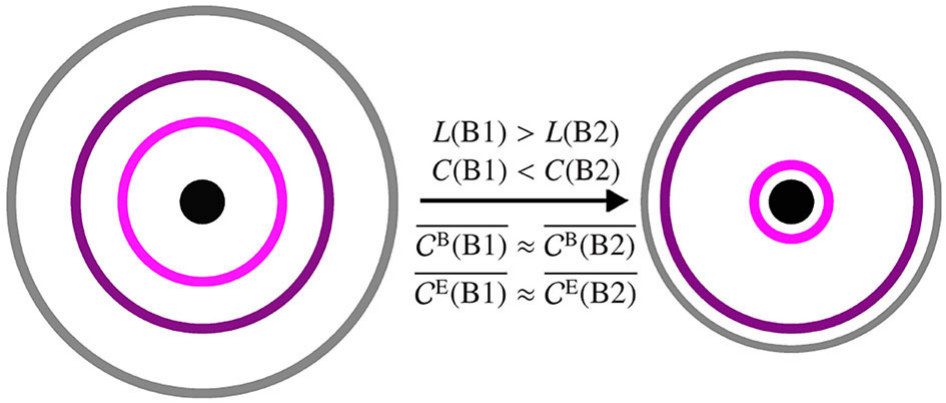
Schematic of a spatial network prior to stimulation (phase B1, **left**) and after stimulation (phase B2, **right**). The network is separated into different areas based on global clustering coefficient *C* and average shortest path length *L*: boundary (gray outermost ring), periphery (purple and pink rings in the middle), core (black innermost ring/circle). The larger the radius of a ring the higher the average shortest path length and the smaller the average clustering coefficient of vertices in this area. The closer two rings the more clustered are the vertices in the two areas relative to each other. Averaged centralities ($\bar{C^{\text{B}}}$ and $\bar{C^{\text{E}}}$) are not affected by the stimulation.

The differential, taVNS-mediated modifications of local and global topological characteristics of evolving functional brain networks may appear contradictory, at first glance. This inconsistency, however, can be resolved when considering the following model of a stimulation-induced *stretching* and *compression* of the network (see Figure 4; cf. Fruengel et al., 2020), which may be due to some nonlinear mechanism. The stimulation-induced increase of global clustering coefficient and decrease of average shortest path length points to an, on average, global compression of the evolving functional brain network. However, since spatially and temporally averaged centrality values remained constant, the compression is compensated by some stretching effect. Even though the functional brain network is a fully connected network and has no actual spatial embedding, we can nevertheless visualize (at least conceptually) the stretching- and compression-mediated network modifications by making use of the definitions of path and strength (i.e., weighted degree) in our network approach. To this end, we consider a spatial network scheme in which we separate the network in different areas based on their different global properties (global clustering coefficient and average shortest path length). In Figure 4, these areas—referred to here as core, periphery, and boundary—are represented by rings. The network core contains vertices with highest strength, and we visualize this property as spatial closeness, being the innermost ring with the smallest radius. The network boundary, represented by the outermost ring, contains vertices that are positioned farthest from the core. Eventually, the network periphery (purple and pink rings) comprises everything in between core and boundary. Now, as a result of the stimulation the network periphery does not change homogeneously. The observed increase of global clustering coefficient and decrease of average shortest path length, together with an unaltered average centrality, can be explained by a compression of the network boundary as well as by a partly compression of the network periphery (colored rings). Hence the network boundary gets closer to the network core, leading to the decrease in average shortest path length. The network boundary also gets closer to a part of the network periphery (gray ring gets closer to purple ring), while on the other hand parts of the network periphery get closer to the network core (pink ring gets closer to black innermost ring). This represents the increase in the global clustering coefficient. The network periphery in itself, however, is stretching (purple and pink rings get further apart) compensating for the global compression, while retaining the path structure prior to stimulation. This then results in an unaltered average eigenvector and betweenness centrality as well as an unaltered ranking. We conjecture that these topology-modifying stretching and compression effects also affect assortativity and synchronizability of the network, thereby enhancing its robustness and stability. The implications of these global and local stimulation-induced network modifications for (patho-)physiological brain functioning, however, remain to be shown. Nevertheless, in the future, tracking network characteristics could be utilized for monitoring stimulation-based interventions in diverse CNS disorders (Helmstaedter et al., 2021).

## 5. Conclusion

We demonstrated that short-term taVNS modifies local and global topological properties as well as stability and robustness properties of evolving functional brain networks, which is in line with the prevalent view of a global-acting mode of action of taVNS. This mode of action, being spatially unspecific on a local network scale, can be explained with the here proposed model of a stimulation-related stretching and compression of functional brain networks.

## Data Availability Statement

The datasets presented in this article are not readily available because they contain information that could compromise the privacy of research participants. Requests to access the datasets should be directed to klaus.lehnertz@ukbonn.de.

## Ethics Statement

The studies involving human participants were reviewed and approved by the ethics committee of the University of Bonn. The patients/participants provided their written informed consent to participate in this study.

## Author Contributions

All authors conceived the research project and wrote the paper. All authors contributed to the article and approved the submitted version.

## Conflict of Interest

RvW received once a fee for lecture from Cerbomed in 2016. The remaining authors declare that the research was conducted in the absence of any commercial or financial relationships that could be construed as a potential conflict of interest.

## Publisher's Note

All claims expressed in this article are solely those of the authors and do not necessarily represent those of their affiliated organizations, or those of the publisher, the editors and the reviewers. Any product that may be evaluated in this article, or claim that may be made by its manufacturer, is not guaranteed or endorsed by the publisher.
