# Supplementary material for: Impact of Transcutaneous Auricular Vagus Nerve Stimulation on Large-Scale Functional Brain Networks: From Local to Global
Source: Front Physiol. 2021 Aug 20;12:700261. doi: 10.3389/fphys.2021.700261 (PMC8417898; doi:10.3389/fphys.2021.700261)
Supplement: Supplementary file 1 [file Data_Sheet_1.pdf]

Supplementary Material

APPENDIX

A1 - Synchronization index

The synchronization index *mean phase coherence* (Mormann et al., 2000) provides an estimate for the strength of interactions between the dynamics of brain regions n and m ($(n,m) \in \{1,\ldots,N\}$). It is defined as:

$$r_{nm} := \left| \frac{1}{T} \sum_{j=0}^{T-1} e^{i(\Phi_n(j)-\Phi_m(j))} \right|,$$

with the number of data points T. Φ_n and Φ_m denote time series of instantaneous phases that we derived from the respective EEG time series using the Hilbert transform. r_{nm} falls within the range $[0,1]$, where $r_{nm} = 1$ indicates fully phase-synchronized systems, while $r_{nm} = 0$ indicates no phase synchronization. Note that with this analytic signal approach the instantaneous frequency relates to the predominant frequency in the Fourier spectrum (Boashash, 1992) (particularly in case of two or more superimposed oscillatory components). Since the predominant frequency may fluctuate in the EEG time series, the instantaneous frequency can vary rhythmically around the predominant frequency which results in spurious estimates of the instantaneous phase. Taking the temporal average can reduce such effects. From an electrophysiological point of view, we consider it more reasonable to investigate adaptively (e.g., via the Hilbert transform) interactions between predominant rhythms in the EEG rather than looking at interactions in some a priori fixed frequency bands (e.g., via wavelet transform) for which there is no power in the time series (Osterhage et al., 2007).

A2 - Network characteristics

Network characteristics considered here are based on either one or on a combination of the graph-theoretical concepts of degree, strength, and paths. Generally, we refer to the sets of vertices and edges constituting a network with $\mathcal{V}$ and $\mathcal{E}$, and $N = |\mathcal{V}|$ and $E = |\mathcal{E}|$ denote the number of vertices and edges, respectively. While the degree κ_n of vertex n describes the number of neighboring vertices connected to it, the strength s_n of that vertex describes the sum of the edge weights of all edges connected to the vertex; $s_n := \sum_{m=1}^{N} W_{nm}^{(\mathrm{v})}$. Here, $W_{nm}^{(\mathrm{v})}$ is an entry in the symmetric weighted vertex adjacency matrix $\mathbf{W}^{(\mathrm{v})} \in \mathbb{R}_+^{N\times N}$ and denotes the weight of the edge connecting vertices n and m. To exclude self-loops we also define $W_{nn}^{(\mathrm{v})} := 0 \,\forall\, n$ with $n \in \{1,\ldots,N\}$. A path between two vertices or edges in a network contains all edges that have to be traversed in order to get from one vertex/edge to the other. The length d_{nm} of a shortest path P between vertices or edges n and m in a weighted network is defined as the sum of the inverse weights of edges along this path (Freeman, 1979). In case of adjacent edges, i.e., edges connected by a single vertex, we define $d_{nm} := 0$.

A2.1 - Local network characteristics

The importance of network constituents (vertices and edges) can be assessed with the concept of centrality that allows for various interpretations (Lü et al., 2016). Centrality indices take into account the different roles network constituents play in a network. Here, we concentrate on the path-based concept of

betweenness centrality and the strength-based concept of eigenvector centrality.

Betweenness centrality—Following Newman (2001); Barrat et al. (2004); Wang et al. (2008); Opsahl et al. (2010); Bröhl and Lehnertz (2019), vertex betweenness centrality (of vertex k) can be defined as

$$\mathcal{C}_{\mathrm{v}}^{\mathrm{B}}(k) := \frac{2}{(N-1)(N-2)} \sum_{k \neq n \neq m} \frac{q_{nm}(k)}{Q_{nm}},$$

where $\{k, n, m\} \in \mathcal{V}$, and $q_{nm}(k)$ is the number of shortest paths between vertices n and m running through vertex k. Q_{nm} is the total number of shortest paths between vertices n and m.

Similarly, edge betweenness centrality (of edge k) can be defined as (Freeman, 1977; Girvan and Newman, 2002)

$$\mathcal{C}_{\mathrm{e}}^{\mathrm{B}}(k) := \frac{2}{N(N-1)} \sum_{n \neq m} \frac{q_{nm}(k)}{Q_{nm}},$$

where $k \in \mathcal{E}$, $\{n, m\} \in \mathcal{V}$, $q_{nm}(k)$ is the number of shortest paths between vertices n and m running through edge k, and Q_{nm} is the total number of shortest paths between vertices n and m.

A network constituent k is the more important, the more commonly this constituent is part of the shortest path between every possible pair of vertices, excluding the pairs with vertex k.

Eigenvector centrality—Following Bröhl and Lehnertz (2019) (and references therein), vertex eigenvector centrality (of vertex k) is defined as the kth entry of the eigenvector $\vec{v}$ corresponding to the dominant eigenvalue $\lambda_{\max}$ of matrix $\mathbf{M}$, which we derive from the eigenvector equation $\mathbf{M}\vec{v} = \lambda\vec{v}$ using the power iteration method:

$$\mathcal{C}_{\mathrm{v}}^{\mathrm{E}}(k) := \frac{1}{\lambda_{\max}} \sum_{l} M_{kl}\, \mathcal{C}_{\mathrm{v}}^{\mathrm{E}}(l),$$

with $\{k, l\} \in \mathcal{V}$. Here $\mathbf{M}$ denotes the weighted vertex adjacency matrix $\mathbf{W}^{(\mathrm{v})}$.

Analogously, edge eigenvector centrality (of edge k) is defined as

$$\mathcal{C}_{\mathrm{e}}^{\mathrm{E}}(k) := \frac{1}{\lambda_{\max}} \sum_{l} M_{kl}\, \mathcal{C}_{\mathrm{e}}^{\mathrm{E}}(l),$$

with $\{k, l\} \in \mathcal{E}$. Here $\mathbf{M}$ denotes the weighted edge adjacency matrix $\mathbf{W}^{(\mathrm{e})} \in \mathbb{R}_{+}^{E \times E}$ whose entries $W_{nm}^{(\mathrm{e})}$ are assigned the average weight of edges n and m if these edges are connected to a same vertex, and 0 otherwise. As above, we define $W_{nn}^{(\mathrm{e})} := 0 \,\forall\, n$ with $n \in \{1, \ldots, E\}$.

A network constituent k is important if its adjacent constituents of the same type are also important.

A2.2 - Global network characteristics

Global clustering coefficient—Following Onnela et al. (2005); Ansmann and Lehnertz (2012), the global clustering coefficient of a weighted network consisting of N vertices can be defined as:

$$C := \binom{N}{3}^{-1} \sum_{n=1}^{N} \sum_{m=1}^{n-1} \sum_{l=1}^{m-1} \frac{\sqrt[3]{W_{nm}^{(\mathrm{v})} W_{ml}^{(\mathrm{v})} W_{ln}^{(\mathrm{v})}}}{\max\left(\mathcal{W}\right)},$$

with the collection of all edge weights $\mathcal{W} := \left\{ W_{nm}^{(\mathrm{v})} \,\middle|\, 1 \leq n < m \leq N \right\}$.

Average shortest path length—For a weighted network, one can define the length of an edge as the inverse of the weight of that edge. If we exclude the path from one vertex to itself from the mean, the averages shortest path length can be defined as (Ansmann and Lehnertz, 2012):

$$L := \binom{N}{2}^{-1} \sum_{n=1}^{N} \sum_{m=1}^{n-1} \min_{l} \min_{P \in \mathcal{P}_{nm}^{l}} \sum_{k=1}^{l-1} W_{P_k P_{k+1}}^{(\mathrm{v})-1},$$

where $\mathcal{P}_{nm}^{l} := \left\{ P \in \{1, \ldots, N\}^{l} \middle| P_1 = n, P_l = m \right\}$ is the set of all paths of binary length l from n to m, and $W_{nm}^{(\mathrm{v})-1} = \infty$ if $W_{nm}^{(\mathrm{v})} = 0$.

Assortativity—This characteristic quantifies whether vertices preferentially connect to vertices with a similar degree (Newman, 2002; Bialonski and Lehnertz, 2013). For binary networks, assortativity A is defined as the correlation coefficient over $\{(\kappa_n, \kappa_m) | G_{nm} = 1, 1 \leq n, m \leq N\}$:

$$A := \left(2K_1 \sum_{n=1}^{N} \sum_{m=1}^{n-1} G_{nm} \kappa_n \kappa_m - K_2^2 \right) / \left(K_1 K_3 - K_2^2 \right),$$

with $K_q := \sum_{l=1}^{N} \kappa_l^q$. G_{nm} are entries of the binary adjacency matrix $\mathbf{G} \in \{0,1\}^{N \times N}$ and κ_n is the degree of vertex n. To obtain $\mathbf{G}$, we transform the weighted vertex adjacency matrix $\mathbf{W}^{(\mathrm{v})}$ with a threshold approach (Lehnertz et al., 2017):

$$G_{nm} = \begin{cases} 1 & \text{if } W_{nm}^{(\mathrm{v})} > \theta, n \neq m \\ 0 & \text{otherwise.} \end{cases}$$

The threshold θ is chosen such, that the resulting binary network is connected and has a fixed number of edges (here: $E = 80$). A is confined to the interval $[-1, 1]$ by definition. Positive (negative) values of A indicate an assortative (disassortative) network.

Synchronizability—The stability of the globally synchronized state of a network can be characterized by the *eigenratio* of the network which is defined as $S := \lambda_N / \lambda_2$ (Barahona and Pecora, 2002; Atay et al., 2006). Here, λ_N denotes the largest eigenvalue of the Laplacian $\mathcal{L}$ of the network ($\mathcal{L}_{nm} := \kappa_n \delta_{nm} - G_{nm}$ for binary networks and $\mathcal{L}_{nm} := s_n \delta_{nm} - W_{nm}^{(\mathrm{v})}$ for weighted networks, where δ is the Kronecker delta and s_n denotes the strength of vertex n). λ_2 denotes the second smallest eigenvalue of the Laplacian (the smallest being 0). Given some vertex dynamics, the higher S the less stable is the synchronized state of the network. This interpretation crucially depends on the definition of S (note that other definitions, e.g. $S := \lambda_2 / \lambda_N$ were proposed in the literature).

REFERENCES

Ansmann, G. and Lehnertz, K. (2012). Surrogate-assisted analysis of weighted functional brain networks. *J. Neurosci. Methods* 208, 165–172. doi:10.1016/j.jneumeth.2012.05.008

Atay, F. M., Bıyıkoğlu, T., and Jost, J. (2006). Network synchronization: Spectral versus statistical properties. *Physica D* 224, 35–41. doi:10.1016/j.physd.2006.09.018

Barahona, M. and Pecora, L. M. (2002). Synchronization in small-world systems. *Phys. Rev. Lett.* 89, 54101. doi:10.1103/PhysRevLett.89.054101

Barrat, A., Barthélemy, M., Pastor-Satorras, R., and Vespignani, A. (2004). The architecture of complex weighted networks. *Proc. Natl. Acad. Sci. U.S.A.* 101, 3747–3752. doi:10.1073/pnas.0400087101

Bialonski, S. and Lehnertz, K. (2013). Assortative mixing in functional brain networks during epileptic seizures. *Chaos* 23, 033139. doi:10.1063/1.4821915

Boashash, B. (1992). *Time frequency signal analysis: methods and applications* (Melbourne: Longman Cheshire)

Bröhl, T. and Lehnertz, K. (2019). Centrality-based identification of important edges in complex networks. *Chaos* 29, 033115. doi:10.1063/1.5081098

Freeman, L. C. (1977). A set of measures of centrality based on betweenness. *Sociometry* 40, 35–41

Freeman, L. C. (1979). Centrality in social networks: Conceptual clarification. *Soc. Networks* 1, 215–239. doi:10.1016/0378-8733(78)90021-7

Girvan, M. and Newman, M. E. J. (2002). Community structure in social and biological networks. *Proc. Natl. Acad. Sci. U.S.A.* 99, 7821–7826. doi:10.1073/pnas.122653799

Lehnertz, K., Geier, C., Rings, T., and Stahn, K. (2017). Capturing time-varying brain dynamics. *EPJ Nonlin. Biomed. Phys.* 5, 2

Lü, L., Chen, D., Ren, X.-L., Zhang, Q.-M., Zhang, Y.-C., and Zho, T. (2016). Vital nodes identification in complex networks. *Phys. Rep.* 650, 1–63. doi:http://dx.doi.org/10.1016/j.physrep.2016.06.007

Mormann, F., Lehnertz, K., David, P., and Elger, C. E. (2000). Mean phase coherence as a measure for phase synchronization and its application to the EEG of epilepsy patients. *Physica D* 144, 358–369. doi:10.1016/S0167-2789(00)00087-7

Newman, M. E. J. (2001). Scientific collaboration networks. II. Shortest paths, weighted networks, and centrality. *Phys. Rev. E* 64, 016132. doi:10.1103/PhysRevE.64.016132

Newman, M. E. J. (2002). Assortative mixing in networks. *Phys. Rev. Lett.* 89, 208701. doi:10.1103/PhysRevLett.89.208701

Onnela, J. P., Saramäki, J., Kertész, J., and Kaski, K. (2005). Intensity and coherence of motifs in weighted complex networks. *Phys. Rev. E* 71, 065103. doi:10.1103/PhysRevE.71.065103

Opsahl, T., Agneessens, F., and Skvoretz, J. (2010). Node centrality in weighted networks: Generalizing degree and shortest paths. *Soc. Networks* 32, 245–251. doi:10.1016/j.socnet.2010.03.006

Osterhage, H., Mormann, F., Staniek, M., and Lehnertz, K. (2007). Measuring synchronization in the epileptic brain: A comparison of different approaches. *Int. J. Bifurcation Chaos Appl. Sci. Eng.* 17, 3539–3544

Wang, H., Hernandez, J. M., and Van Mieghem, P. (2008). Betweenness centrality in a weighted network. *Phys. Rev. E* 77, 046105
